# LGBTQ+ OLDER ADULT COUPLES' EXPERIENCES OF MINORITY STRESS BY SERVICE PROVIDERS

**DOI:** 10.1093/geroni/igac059.167

**Published:** 2022-12-20

**Authors:** Sara Bybee, Kristin Cloyes, Kathi Mooney, Katherine Supiano, Brian Baucom, Lee Ellington

**Affiliations:** University of Utah, Salt Lake City, Utah, United States; Oregon Health & Science University, Portland, Oregon, United States; University of Utah College of Nursing, Salt Lake City, Utah, United States; University of Utah, Salt Lake City, Utah, United States; University of Utah Department of Psychology, Salt Lake City, Utah, United States; University of Utah and Huntsman Cancer Institute, Salt Lake City, Utah, United States

## Abstract

This study explored LGBTQ+ older adult couples’ experiences of minority stress with service providers and effects on their relationships. Twelve LGBTQ+ cancer patient-partner couples (N = 24) completed surveys assessing demographics, stress, and health, and participated in dyadic semi-structured interviews. Descriptive statistics summarized demographic characteristics. Interview data were content analyzed to identify sources of minority stress. Participants were aged 50.9 years on average (SD = 9.9, R =32-70), mostly white (21, 87.5%), and had been together for 19.1 years (SD = 9.9, R = 9-44). Common minority stress sources included derogatory language, belittling comments, heteronormativity, and cisnormativity in routine healthcare, cancer care, and legal services. Couples attributed relationship strength and durability as mitigating negative effects of stress; they described feeling closer, stronger, and more confident in their relationships. Some couples denied experiences of minority stress by service providers and these couples ascribed their equitable care to their geographic location.

